# Thank you to our reviewers of 2025

**DOI:** 10.1002/epi4.70219

**Published:** 2026-02-13

The Editors of Epilepsia Open extend their sincere thanks to our associate editors, editorial board members, and the reviewers listed below for their invaluable service during 2025. Their thoughtful evaluations, expertise, and commitment of time are fundamental to maintaining the scientific rigor and quality of the journal. We are deeply grateful for their contributions in advancing epilepsy research and supporting the peer review process.

Merab Kokaia, PhD, Prof. Emeritus

Editor‐in‐Chief, *Epilepsia Open*

merab.kokaia@med.lu.se

Piero Perucca, MD, PhD, Professor

Deputy Editor, *Epilepsia Open*

***≥6 reviews, **≥3–5 reviews, *1–2 reviews

Massimo Cossu***

Bruce Hermann***

David McArthur***

Marco Medina***

Cameron Metcalf***

Heidi Munger Clary***

Hiroki Nariai***

Tomonori Ono***

Daichi Sone***

Catherine Stamoulis***

Pasquale Striano***

Dènahin Toffa***

Kette Valente***

Johan Zelano***

Laura Abraira**

Heather Angus‐Leppan**

Eishi Asano**

Christoph Baumgartner**

Albert Becker**

Alessia Cafaro**

Lixin Cai**

Laura Canafoglia**

Maria Roberta Cilio**

Erin Conrad**

Arthur Cukiert**

Luca De Palma**

Jie Deng**

Giancarlo Di Gennaro**

Raffaele Dubbioso**

Edward Faught**

Edoardo Ferlazzo**

Alessandro Ferretti**

Anthony Fine**

Robert Fisher**

Emma Foster**

Jacqueline French**

Alon Friedman**

Milena Gandy**

Jan Gorter**

Anne Hagemann**

Haslina Hashim**

Martin Holtkamp**

Masaki Iwasaki**

Vasileios Kokkinos**

Naoto Kuroda**

Simona Lattanzi**

Chou‐Ching Lin**

Yicong Lin**

Oreste Marsico**

Janet Mifsud**

James Mills**

Francesco Pasini**

Ronit Pressler**

Vineet Punia**

Mark Quigg**

Georgia Ramantani**

Cristina Reschke**

Antonella Riva**

Roberta Roberti**

Luisa Rocha**

Jitendra Sahu**

Gabriele Saretzki**

Yonatan Serlin**

Renée Shellhaas**

Krzysztof Smolik**

Nicola Specchio**

Andrea Stabile**

Joel Stein**

Ana Suller‐Marti**

Olafur Sveinsson**

Payam Tabaee Damavandi**

James Tao**

Laura Tassi**

Delphine Taussig**

César Teixeira**

Roland Thijs**

Xin Tian**

Mario Tombini**

Michel van Putten**

Libor Velisek**

Richard Verrier**

Aglaia Vignoli**

Vicente Villanueva**

Yu Wang**

Zhong Irene Wang**

Yaroslav Winter**

Ye Wu**

Christina Briscoe Abath*

Walid Abdel Ghany*

Taylor Abel*

Nathan Absalom*

Sophie Adler*

José Ángel Aibar*

Emel Akarsu*

Ozlem Akman*

Halley Alexander*

Dima Alhomsi*

Jane Allendorfer*

Itziar Alonso*

Faisal Alotaibi*

Luís‐Jorge Amaral*

Danielle Andrade*

Pedro Andrade*

Ralph Andrzejak*

Noritoshi Arai*

Caren Armstrong*

Melody Asukile*

Carolina Ferreira Atuesta*

Kurtis Auguste*

Jerome Aupy*

Stéphane Auvin*

Marina Azevedo*

Jerome Badaut*

Mark Baker*

Alice Ballerini*

Dipika Bansal*

Carmen Barba*

Melissa Barker‐Haliski*

Emanuele Bartolini*

Lisa Bateman*

Giulia Battaglia*

Tobias Bauer*

Marcel Bausch*

Vincenzo Belcastro*

Selim Benbadis*

Sándor Beniczky*

Tuba Enise Benli*

Mark Bennett*

Felix Benninger*

Danilo Bernardo*

Federica Bertaso*

Astrid Bertsche*

Andrea Bianconi*

Christian Bien*

Johan Bjellvi*

Leah Blank*

Hal Blumenfeld*

McKenna Bolger*

Sam Booker*

Angelique Bordey*

Karin Borges*

Ingo Borggrafe*

Magdalena Bosak*

Justin Botterill*

Stephen Bowden*

Isabella Brambilla*

Christian Brandt*

David Brenner*

Amy Brewster*

Stephen Briggs*

Serena Broggi*

Andreas Brunklaus*

Paulo Caceres Guido*

Gaetano Cantalupo*

Liqin Cao*

Simona Cappelletti*

Roberto Caraballo*

Evelina Carapancea*

Jaime Carrizosa*

Andrea Cascianelli*

Pablo Casillas Espinosa*

Carlos Cepeda*

Giulia Sofia Cereda*

Mackenzie Cervenka*

Aswin Chari*

Rui Chen*

Yotin Chinvarun*

Catherine Christian‐Hinman*

Ana Carolina Coan*

Matthew Coleman*

Christopher Colwell*

Kelly Conner*

Daniëlle Copmans*

Antonietta Coppola*

Mark Cunningham*

Alice Dainelli*

Isabella D'Andrea*

Felice D'Arco*

Sandhitsu Das*

Kathryn Davis*

Marco de Curtis*

Barbara Decker*

Valentina De Giorgis*

Ahmad Reza Dehpour*

Ennio Del Giudice*

Francesco Deleo*

Antoine Depaulis*

Nagita Devi*

Rossella Di Sapia*

Jacopo DiFrancesco*

Nikolay Dokholyan *

Fedele Dono*

Gianluca D'Onofrio*

Derek Doss*

Laurie Douglass*

Mattias Duempelmann*

Gian Marco Duma*

Felix Ekman*

Riëm El Tahry*

Srinivasan Ekambaram*

Ayse Deniz Elmali*

Sofia Eriksson*

Camilo Espinosa‐Jovel*

Jessica Falco‐Walter*

Merce Falip*

Odile Feys*

Niels Foit*

Tom Foley*

Si Lei Fong*

Thomas Foutz*

Jonah Fox*

Daniel Friedman*

Tamar Gachechiladze*

William Gaetz*

Zsolt Gáll*

Marian Galovic*

Taneeta Ganguly*

Elena Gardella*

David Garnica Agudelo*

Nicolas Gaspard*

Sara Gasparini*

Gopisankar Mohanannair Geethadevi*

Antonio Giulio Gennari*

Joanna Gesche*

Bruria Gidoni Ben Zeev*

Lucio Giordano*

Giada Giovannini*

Vadym Gnatkovsky*

Zachary Goldblum*

Rianne Goselink*

Sushma Goyal*

Tiziana Granata*

Bin Gu*

Milena Guazzi*

James Gugger*

Michelle Guignet*

Gunay Gul*

MV Gule*

Jiahao Guo*

Roy Haast*

Lena Habermehl*

Michael Hadler*

Sugondo Hadiyoso*

Alexander Haglund*

Amy Halliday*

Marla Hamberger*

Gerben van Hameren*

Kevin Hampel*

Zulfi Haneef*

Aurelie Hanin*

Martin Hardmeier*

Rebekah Harris*

William Harris*

Hans Hartmann*

Itaru Hayakawa*

Xiaosong He*

Michael Hildebrand*

Chloe Hill*

Martin Hirsch*

Manisha Holmes*

Annette Holth Skogan*

Naoto Hoshi*

Saman Hosseini Ashtiani*

David Howard*

Chin‐Wei Huang*

Shaun Hussain*

Lukas Imbach*

Dorien Imberechts*

Tamjid Imtiaz*

Takao Inoue*

Mary Iype*

Sobodan Jankovic*

Jacek Jaworski*.

Hongbae Jeong*

Fuchang Jiang*

Hennric Jokeit*

Charuta Joshi*

Suchitra Joshi*

Mark Kaddumukasa*

Andres Kanner*

Shefali Nakul Karkare*

Victor Karpychev*

Dorothee Kasteleijn‐Nolst Trenite*

Anna‐Maria Katsarou*

Lesley Kaye*

Meghann Kelly*

Wesley Kerr*

Ankit Khambhati*

Ramin Khatami*

Yun Seo Kim*

Lohith Kini*

Johanna Kissler*

Susanne Knake*

Robert Knowlton*

Matthias Koepp*

Ramesh Konanki*

Otar Koniashvili*

Eric Kossoff*

Sanjeev Kothare*

Katarzyna Kotulska*

Richard Kovacs*

Günter Krämer*

Ruzica Kravljanac*

Balu Krishnan*

David Krysl*

Hana Kubova*

Harley Kurata*

Churl‐Su Kwon*

Lieven Lagae*

Cedric Laloyaux*

Jacopo Lanzone*

Daniel Lapid*

Fiona LeBeau*

Alan Leviton*

Qiang Li*

Tianfu Li*

Xiaojin Li*

Jianxiang Liao*

Laura Librizzi*

Kheng Seang Lim*

Christos Panagiotis Lisgaras*

Janine Liu*

Lili Long*

Iscia Lopes‐Cendes*

David Loring*

Anne Lortie*

Huaxia Luo*

Annika Lüttjohann*

Priyanka Madaan*

Francesca Madia*

Rama Maganti*

Chiara Malmberg*

Michael Malter*

Giorgi Mamardashvili *

Mohammad Amin Manavi*

Maria Margherita Mancardi*

Mark Manford*

Massimo Mantegazza*

Christopher Martinez Aguirre*

Marta Maschio*

Federico Mason*

Shavonne Massey*

Andrey Mazarati*

Adolfo Mazzeo*

Carrie McDonald*

Tanya McDonald*

Aileen McGonigal*

Oriano Mecarelli*

Davide Mei*

Christian Meisel*

Stefano Meletti*

Mattia Mercier*

Mathieu Milh*

Berge Minassian*

Usha Kant Misra*

Ahsan Moosa*

Victoria Morgan*

Solomon Moshe*

Wenzhu Mowrey*

Susanne Mueller*

Marco Mula*

Sandra Munteanu*

Jamil Muradov*

Nobukazu Nakasato

Astrid Nehlig*

Lenycia de Cassya Lopes Neri*

John‐Paul Nicolo*

Vanesa Nieto‐Estevez*

Takuji Nishida*

Margherita Nosadini*

Ayako Ochi*

Hirokazu Oguni*

Masayoshi Oguri*

William Ojemann*

Karmele Olaciregui*

Ingrind Olsson Lindberg*

Chima Oluigbo*

Suyi Ooi*

Sandra Orozco‐Suarez*

Alessandro Orsini*

Juan Fernando Padin Nogueira*

Trudy Pang*

Kang Min Park*

Angelo Pascarella*

Grazia Pastorino*

Ekaterina Pataraia*

Jacob Pellinen*

Piero Perucca*

Gabriela Pesántez Ríos*

Marta Piccioli*

Nicola Pietrafusa*

Marta Pietruszka*

Luciana Pimentel‐Silva*

Francesco Pisani*

Laura Rosa Pisani*

Sebastian Pollandt*

Julia Pottkaemper*

Prakash Poudel*

Dario Pruna*

Suresh Pujar*

Jan Pukropski*

Isha Puntabekar*

Citra Raditha*

Ali Rafati*

Shervin Rahimpour*

Alberto Javier Ramos*

Stefan Rampp*

Teresa Ravizza*

Margie Ream*

Christopher Reid*

Colin Reilly*

Elisabeth Reus*

Anny Reyes*

Xiana Rodríguez‐Osorio*

Antonino Romeo*

Alexander Rotenberg*

Guido Rubboli*

Clio Rubinos*

Emilio Russo*

Philippe Ryvlin*

Arushi Saini*

Pauline Samia*

Josemir Sander*

Daniel San‐Juan*

Raman Sankar*

Cesar Santana‐Gomez*

Bina Santoro*

Peter Sarnacki*

Morris Scantlebury*

Olaf Schijns*

Friedhelm Schmitt*

Andreas Schulze‐Bonhage*

Pokmeng See*

Desiree R. Seib*

Udaya Seneviratne*

Leigh Sepeta*

Pedro Serrano‐Castro*

Oleksii Shandra*

Suvasini Sharma*

Beth Sheidley*

Khaled Shewey*

Hideaki Shiraishi*

Daniel Shrey*

Meneka Sidhu*

Mary Silvia*

Nishant Sinha*

Deepa Sirsi*

Shobi Sivathamboo*

Stuart Smith*

Ashwini Sri Hari*

Carl Stafstrom*

Alena Stasenko*

Mirja Steinbrenner*

Bernhard Steinhoff*

Manuela Stella*

Claude Steriade*

David Steven*

Adam Strzelczyk*

Joseph Sullivan*

Marte Syvertsen*

Charles Szabo*

Maurizo Taglialatela*

Masanori Takeoka*

Dimitris N. Tatakis*

Gaetano Terrone*

John Thomas*

Moussa Toudou Daouda*

Chahnez Triki*

Marina Trivisano*

Takehiro Uda*

Kazushi Ukishiro*

Juzoh Umemori*

Anthony Umpierre*

Aycan Ünalp*

Angeliki Vakrinou*

Gilles van Luijtelaar*

Nicholas Varvel*

Sandra Vaz*

Alberto Verrotti*

Artur Vetkas*

Federico Vigevano*

Elisa Visani*

Berthold Voges*

Greta Volpedo*

Marec von Lehe*

Felix von Podewils*

Angela Wabulya*

Konrad Wagstyl*

Wasan Wahab*

Qiang Wang*

Xuefeng Wang*

Christopher Whalley*

Abigail White*

Vicky Whittemore*

Michèl Willemsen*

Aislinn J.Williams*

Gavin Winston*

Juri‐Alexander Witt*

Lily Wong‐Kisiel*

Season K. Wyatt‐Johnson*

Yumeng Wang*

Nihal Yıldız*

Nada El Youssef*

Elissa Yozawitz*

Gaetano Zaccara*

Ifrah Zawar*

Junning Zhang*

Wen‐Diao Zhang*

Daniel Zhou*

Ivan Zibrandtsen*

